# Stem girdling indicates prioritized carbon allocation to the root system at the expense of radial stem growth in Norway spruce under drought conditions

**DOI:** 10.1016/j.envexpbot.2017.03.004

**Published:** 2017-06

**Authors:** Walter Oberhuber, Andreas Gruber, Gina Lethaus, Andrea Winkler, Gerhard Wieser

**Affiliations:** a Institute of Botany, Leopold-Franzens-University of Innsbruck, Sternwartestrasse 15, A-6020 Innsbruck, Austria; b Department of Alpine Timberline Ecophysiology, Federal Research and Training Centre for Forests, Natural Hazards and Landscape (BFW), Rennweg 1, A-6020 Innsbruck, Austria

**Keywords:** Carbon allocation, Drought, Girdling, Non-structural carbohydrates, Norway spruce, Radial growth

## Abstract

The early culmination of maximum radial growth (RG) in late spring has been found in several coniferous species in a dry inner Alpine environment. We hypothesized that an early decrease in RG is an adaptation to cope with drought stress, which might require an early switch of carbon (C) allocation to belowground organs. To test this hypothesis, we experimentally subjected six-year-old Norway spruce saplings (tree height: 1.35 m; n = 80 trees) to two levels of soil water availability (watered versus drought conditions) and manipulated tree C status by physically blocking phloem transport at three girdling dates (GD). The influence of C availability and drought on tree growth (radial and shoot growth; root biomass) in response to girdling was analyzed in both treatments. Non-structural carbohydrates (NSCs, soluble sugars and starch) were measured in the stem, root and current leader to evaluate changes in tree C status due to girdling. The main finding was a significant increase in RG of the girdled trees compared to the controls above the girdling zone (UZ). At all girdling dates the RG increase was significantly more intense in the drought-stressed compared with watered trees (*c*. 3.3 and 1.9-fold higher compared with controls in the drought-stressed and watered trees, respectively), most likely indicating that an early switch of C allocation to belowground occurs as an adaptation to maintain tree water status under drought conditions. Reactivation of the cambium after the cessation of its regular activity was detected in UZ in drought-stressed trees, while below the girdling zone no xylem formation was found and the NSC content was strikingly reduced. Irrespective of water availability, girdling before growth onset significantly reduced the progression of bud break (*P* < 0.05) and the length of the current leader shoot by −47% (*P* < 0.01) indicating a reduction in xylem hydraulic conductance, which was corroborated by significantly reduced xylem sap flow (*P* < 0.001). Based on our findings, we conclude that during the growing season drought stress prioritizes an early switch of C allocation to the root system as an adaptation to maintain adequate tree water status in drought-prone environments.

## 1 Introduction

Drought is a major factor affecting growth and wood formation in trees. Both cell division and cell enlargement are affected under drought due to decreased energy supply, loss of cell turgor and impaired enzyme activities (Pallardy, 2008). Several dendroecological studies conducted within dry inner Alpine environments have shown that drought stress in terms of reduced soil water availability in April to June impairs the annual increment of coniferous species (e.g., Zweifel et al., 2006; Pichler and Oberhuber 2007; Schuster and Oberhuber 2013). In addition, determination of the intra-annual dynamics of radial stem growth (RG) revealed that the maximum RG rate already peaked in May through early June, i.e., prior to increases in precipitation and adequate soil water availability during the summer (Gruber et al., 2010; Oberhuber et al., 2014). It is well known that plants can adjust their carbon (C) allocation patterns to optimize resource uptake under prevailing environmental constraints and can increase C allocation to roots in response to drought, i.e., tree species adapted to dry conditions generally have higher root-to-shoot ratios and deeper root systems than species that occur under more mesic environmental conditions (Kozlowski and Pallardy 2002; Brunner et al., 2015). Although C transport to belowground is reduced under severe and prolonged drought possibly due to failure to maintain phloem water status (Sevanto, 2014), several authors reported that under moderate drought assimilate allocation belowground is enhanced (e.g. Meier and Leuschner 2008; Hommel et al., 2016). Hence, the early decrease in stem growth of conifers exposed to low soil water availability can be regarded as an adaptation to maintain adequate tree water status, which might require an early switch of C allocation to the root system and associated mycorrhiza.

The use of manipulative experiments is essential to understand the drivers of plant growth under different environmental conditions. Girdling, i.e., the physical blockage of phloem transport around a tree’s outer circumference, is frequently applied to investigate C relationships (Williams et al., 2000; Maunoury-Danger et al., 2010; De Schepper and Steppe 2011). Due to blocking of the downward translocation of soluble sugars, accumulation and depletion of carbohydrates above and below the girdle, respectively, occur (Daudet et al., 2005; Peuke et al., 2005; Maier et al., 2010), and roots are gradually depleted of their carbohydrate reserves (Högberg et al., 2001; Li et al., 2003). Manipulation of the C status of the stem can reveal the source limitation of RG because RG depends on a continuous supply of carbohydrates (Daudet et al., 2005; Zweifel et al., 2006) and cambial activity is known for its low priority in resource allocation, being preceded by respiration, root growth and storage (Savidge, 2000; Oribe et al., 2003; Polák et al., 2006). Furthermore, carbohydrates are hypothesized to favor growth via the accumulation of osmotically active C compounds, which increase turgor in expanding cells (Deslauriers et al., 2016).

Norway spruce (*Picea abies* (L.) Karst.) is the most widespread coniferous species in the central European Alps, ranging from low elevation to the treeline (Ellenberg and Leuschner, 2010). *Picea abies* is sensitive to soil water availability during the growing season (Lévesque et al., 2013; Leo et al., 2014) and shows early culmination of RG under conditions of soil drought (Oberhuber et al., 2014). In this experimental study, we focused on elucidating the influence of manipulated environmental conditions, i.e., water availability and modified tree C status by girdling at three phenological stages (prior to growth onset, during vigorous radial and shoot growth and after cessation of shoot growth) on tree growth (radial and shoot growth, root biomass). We hypothesized that C allocation to the root system is prioritized over RG particularly under drought, i.e., altered C availability due to phloem blockage at distinct phenological stages differently stimulates RG above the girdling zone at the expense of root growth. The results of this study contribute to the understanding of an early culmination of RG found in coniferous species at drought-prone sites (Oberhuber et al., 2014).

## 2 Materials and methods

### 2.1 Plant material and treatments

The experiment was conducted at the Botanical Garden of the Institute of Botany, University of Innsbruck. In autumn 2013 six-year-old Norway spruce (*Picea abies* (L.) Karst.) trees, previously grown outdoor in a tree nursery, with a stem height of c. 135 cm and a diameter of c. 3.5 cm at a 5-cm stem height were replanted in 80-l containers (filled with fertilized garden soil above a drainage layer at the bottom of the container) and placed in a polytunnel to ensure similar microclimatic conditions for all trees under study. The trees were allowed to recover from transplant shock and adapt to conditions in the containers for one growing season. Starting in mid-March 2015, the trees were subjected to different soil moisture conditions, i.e., watered versus drought-stressed, and were divided into four subsets: the control (no phloem blockage) and a phloem blockage treatment at three girdling dates (GDs) (n = 10 trees in each subset). The experiment included a total of 80 trees (2 environmental settings × 4 subsets × 10 trees = 80 trees).

Air temperature and relative humidity (CS215 temperature and relative humidity sensor, Campbell Scientific, Shepshed, UK) and solar radiation (PhAR; LI-200S Pyranometer Sensor, Campbell Scientific, Shepshed, UK) were continuously measured within the polytunnel at a height of two meters. The soil temperature at 10 cm soil depth (T 107 Temperature Probe, Campbell Scientific, Shepshed, UK) and the volumetric soil water content in the uppermost 30 cm was recorded (CS616 Water Content Reflectometer, Campbell Scientific, Shepshed, UK) for both the watered and drought-stressed trees (n = 10 per treatment). All environmental data were recorded using a CR1000 data logger and three AM 16/32 multiplexers (Campbell Scientific, Shepshed, UK) programmed to record 30-min averages of measurements collected every minute.

### 2.2 Manipulation of C availability by means of double girdling

We applied double girdling to produce three distinct horizontal zones (upper (UZ), middle (MZ) and lower zone (LZ)) with respect to the phloem sap sucrose supply of the stem (De Schepper et al., 2010). While UZ and LZ could still receive carbohydrates from the leaves and roots, respectively, MZ was completely isolated and could only use existing local NSC reserves to maintain metabolism and growth. Two 1–2-cm wide bands of bark (extending to the xylem) were carefully detached from the stem at a height of c. 5 and 15 cm above the soil surface and the xylem tissue was covered with aluminum foil to prevent dehydration. Because concentration of sugars in phloem vary throughout the year and girdling also inhibits transport of shoot-derived growth substances necessary for cambium activity (Larson, 1994), the trees were girdled at three phenological stages: (i) in mid-March 2015 (girdling date [GD] day of the year [doy] 77), i.e., several weeks before bud swelling and the onset of cambial activity; (ii) during vigorous earlywood and shoot growth in mid-May (GD doy 138), and (iii) after cessation of shoot growth in July (GD doy 190). At the latter girdling date RG in drought stressed trees has already stopped for *c*. 6 weeks (cf. Fig. 2).

The ‘pinning method’, i.e., the marking of the cambium by micro-injury using a small needle (diameter *c*. 1 mm), was applied to all three zones to mark GD during the study period (Gričar et al., 2007; Seo et al., 2007). Since the wound tissue separates cells formed before and after pinning, the wound reaction was used for exact dating of wood formation before and after phloem blockage.

### 2.3 Determination of tree phenology, radial stem growth, shoot growth, and root biomass

Phenology of bud break and the growth of the terminal shoot of all trees (n = 10 trees per subset) were recorded in weekly intervals starting in April, whereby four classes were differentiated: 1 = buds slightly swollen, 2 = buds clearly swollen, 3 = needle tissue visible, and 4 = onset of shoot growth. Growth of the terminal shoot was measured using a caliper, and short-term variations were modeled with a Gompertz function (cf. Zeide, 1993) using the non-linear regression procedure included in the Origin software package (OriginLab Corporation, Northampton, MA, USA). Intra-annual dynamics of RG in watered and drought-stressed control trees was monitored by installing automated diameter dendrometers (n = 6 per treatment; type DD-S, Ecomatik, Munich, Germany). Dead outermost layers (periderm) of the bark were slightly removed to ensure close contact with the stem. The belowground biomass (dry weight) of fine roots with a diameter ≤2 mm was measured at the end of the experimental period (late October) for all trees (control and girdled trees).

At the end of the study period stem cross-sections were also collected from all trees from the following locations: the root/shoot transition zone (RS), girdling zones UZ, MZ and LZ approximately 1 cm from the girdling zone to avoid regions with wound responses, 25 and 50 cm above UZ and the current leader. The surface of stem discs was prepared using a sharp razor blade, and ring widths were determined to the nearest 1 μm using an incremental measuring table. Mean RG was calculated from 2 radii per stem section. Dating of the growth zones was accomplished by establishing complete tree-ring series at every girdling zone and by cross-dating the time series visually. To enable comparison of the effects of girdling between the control and girdled trees, RG for each tree was standardized using the ring widths of the year prior to girdling. Standardizing the ring width corrected inherent differences in growth among trees and allowed an assessment of treatment effects between non-girdled and girdled trees over time.

### 2.4 Determination of non-structural carbohydrates (NSCs)

Measurements of starch and soluble sugars (sucrose, glucose, fructose) were performed on watered and drought-stressed trees (n = 10 trees per treatment) using bark samples collected at the time of girdling (GD doy 77, 138, and 190), and from all girdling zones (UZ, MZ, and LZ), the coarse roots and the current leader at the end of the study period in late October or immediately after tree mortality considered as 100% needle browning (cf. Anderegg and Anderegg, 2013). The samples were collected in the morning to minimize the effects of diurnal NSC changes. Within 1 h, enzymes in the samples were denatured by heating the samples in a microwave at 600 W for 90 s (Hoch et al., 2003). Then, the samples were dried at 60 °C to a constant weight, ground into powder (Tissuelyser II, Qiagen, Germany) and stored under dry conditions until they were analyzed.

To bind phenolic substances (e.g., from resins), 0.5 mg of polyvinylpyrrolidone (PVP) was added to *c*. 40 mg of finely ground plant material. Soluble carbohydrates were extracted from the samples twice in 40% (v/v) ethanol for 15 min at 60 °C. After vaporizing the ethanol, the residue of the soluble fraction was resolved in distilled water. Then, catalyzed by hexokinase, glucose was converted into glucose-6-phosphate. The concentration of glucose was determined photometrically at 340 nm, as NADPH^+^ H^+^ formation during the enzymatic conversion of glucose-6-phosphate to gluconate-6-phosphate (Gruber et al., 2012). Sucrose and fructose were enzymatically converted to glucose and glucose-6-phosphate, respectively, which were subsequently quantified as described above. The sucrose and fructose contents were calculated from the difference in the glucose concentrations before and after the respective enzymatic inversion. Starch was measured following solubilization by autoclaving and enzymatic hydrolysis. The insoluble fraction was boiled in water for 3 min and autoclaved for 1 h at 130 °C. After cooling and adjusting the pH using acetate buffer (pH 4.8), AGS (amyloglucosidase) was used to hydrolyze the starch into glucose, which was measured as described above. The enzymatic conversion of the soluble carbohydrates and the photometric determination of NSCs were conducted using a semiautomatic system for photometric testing (Rida Cube Scan analyzer, R-Biopharm, Darmstadt, Germany) and the corresponding enzymatic kits.

### 2.5 Xylem sap flow

Xylem sap flow density (Q_s_) was used as a surrogate for tree transpiration and continuously measured by means of the heat dissipation approach (Granier, 1985) by battery-operated sap flow systems (MI Sapflow Systems Prosa-log; UP, Umweltanalytische Produkte GmbH, Cottbus, Germany). Each system consisted of a three-channel PPROSA-LOG data logger and a constant source for sensor heating. During the growing season (doy 77–270) Q_s_ was recorded in watered and drought-stressed control and girdled (GD doy 138) trees (n = 4-6 trees per treatment). In each study tree, one probe was installed into the outer 2 cm of the sapwood 5–10 cm apart vertically on the north facing side of the stems, 0.2 m aboveground. The upper probe included a heater that was continuously supplied with a constant power of 0.2 W, whereas the lower probe was unheated, remaining at trunk temperature for reference. The temperature difference between the upper heated probe and the lower reference probe was recorded for a certain time interval of 30 min.

For each entire tree Q_s_ (g m^−2^ s^−1^) was calculated from the temperature difference between the two probes (ΔT) relative to the maximum temperature difference (ΔT_m_), which occurs at times of zero flow according to the calibration equation determined by Granier (1985):

$${\text{Q}}_{\text{s}}=119*{\left[\left(\Delta {\text{T}}_{\text{m}}-\Delta \text{T}\right)/\Delta \text{T}\right]}^{1.231}$$

Each night ΔT_m_ was determined and used as a reference for the following day. This assumption of zero sap fluxes seems reasonable as night-time vapor pressure deficits were mostly low and temperature courses of the sensors reached equilibrium most nights, suggesting that refilling of internal reserves was complete. As the heat dissipation approach yields Q_s_ in absolute volume and time units, Q_s_ was scaled up to daily (g cm^−2^ day^−1^) and seasonal (kg cm^−2^) totals.

### 2.6 Statistical analyses

Repeated measure analysis of variance (ANOVA) with post-hoc multiple comparisons (Bonferroni correction) was applied to determine significant differences in the progression of bud break and the dynamics of shoot and root growth and sap flow density among the control and girdled trees. Significant differences in the NSC content were determined by applying the Wilcoxon signed-rank test (SPSS 20; IBM, USA). Student’s independent sample *t*-test was used to determine significant differences among control and girdled trees with respect to RG.

## 3 Results

### 3.1 Environmental conditions and xylem sap flow

**Fig. 1:**
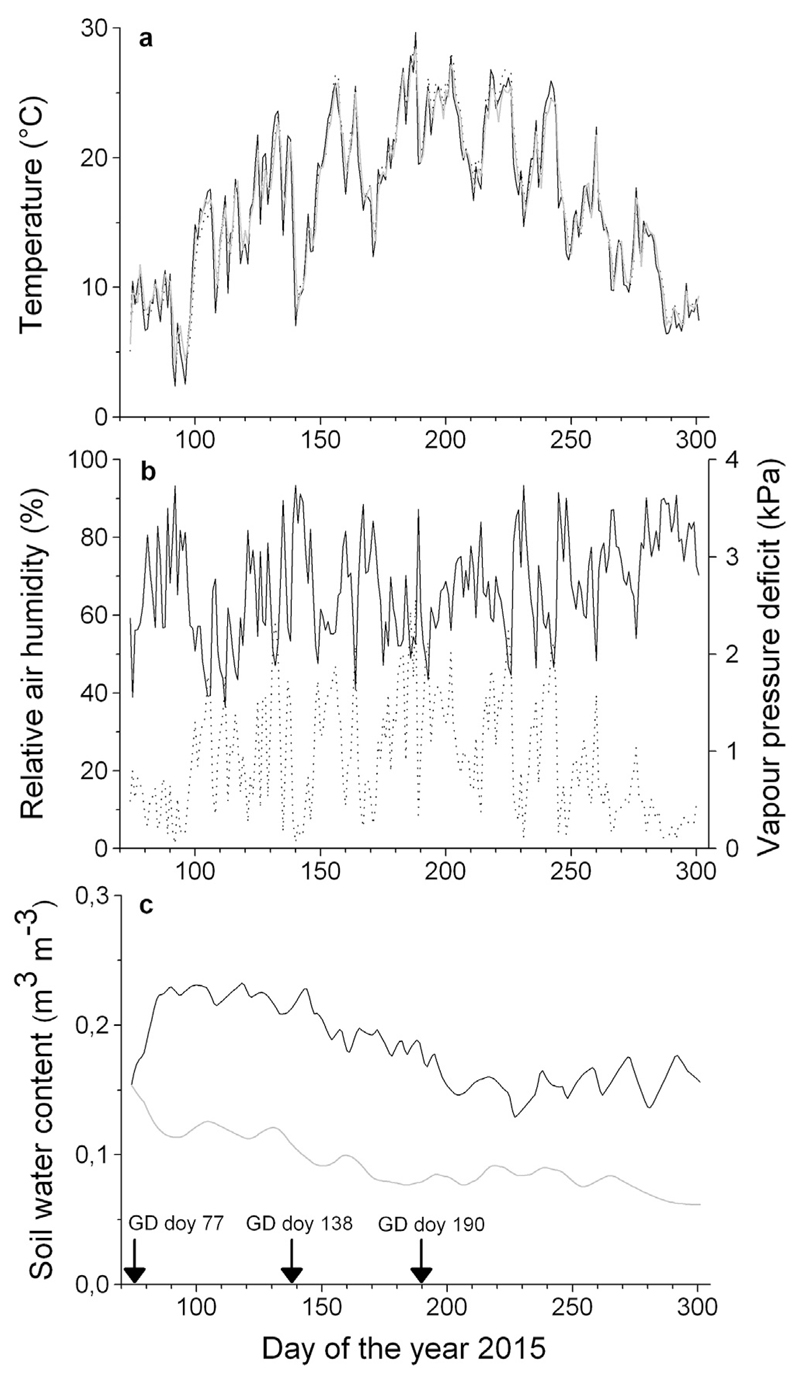
a–c Environmental conditions in the polytunnel during the study period (a) Air temperature (black line) and soil temperature in watered (black dotted line) and drought-stressed (grey line) treatments. (b) Relative air humidity (solid line) and vapor pressure deficit (dotted line). (c) Soil water content (10-day moving averages) in the watered (black line) and drought stressed treatments (grey line). Downward arrows on x-axis indicate the girdling dates (GD).

In the polytunnel, the mean daily air temperature during the study period (April through October) was 18.1 ± 5.6 °C, and the mean daily soil temperature was 18.4 ± 5.3 °C in both treatments (Fig. 1a). The mean daily vapor pressure deficit and relative air humidity ranged between 0.05 and 2.57 kPa (mean = 0.92 ± 0.6 kPa) and 36.4 and 93.4% (mean 65.5 ± 12.8%; Fig. 1b), respectively. The mean daily maximum solar radiation (PhAR) was 1005 μmol m^−2^ s^−1^ (data not shown). At the start of the experiment the volumetric soil water content (SWC) averaged 0.14 ± 0.02 and 0.15 ± 0.02 m^3^ m^−3^ in the watered and drought-stressed treatments, respectively. The mean growing season SWC values were 0.09 ± 0.02 m^3^ m^−3^ in the drought-stressed and 0.18 ± 0.04 m^3^ m^−3^ in the watered containers (Fig. 1c).

**Fig. 2:**
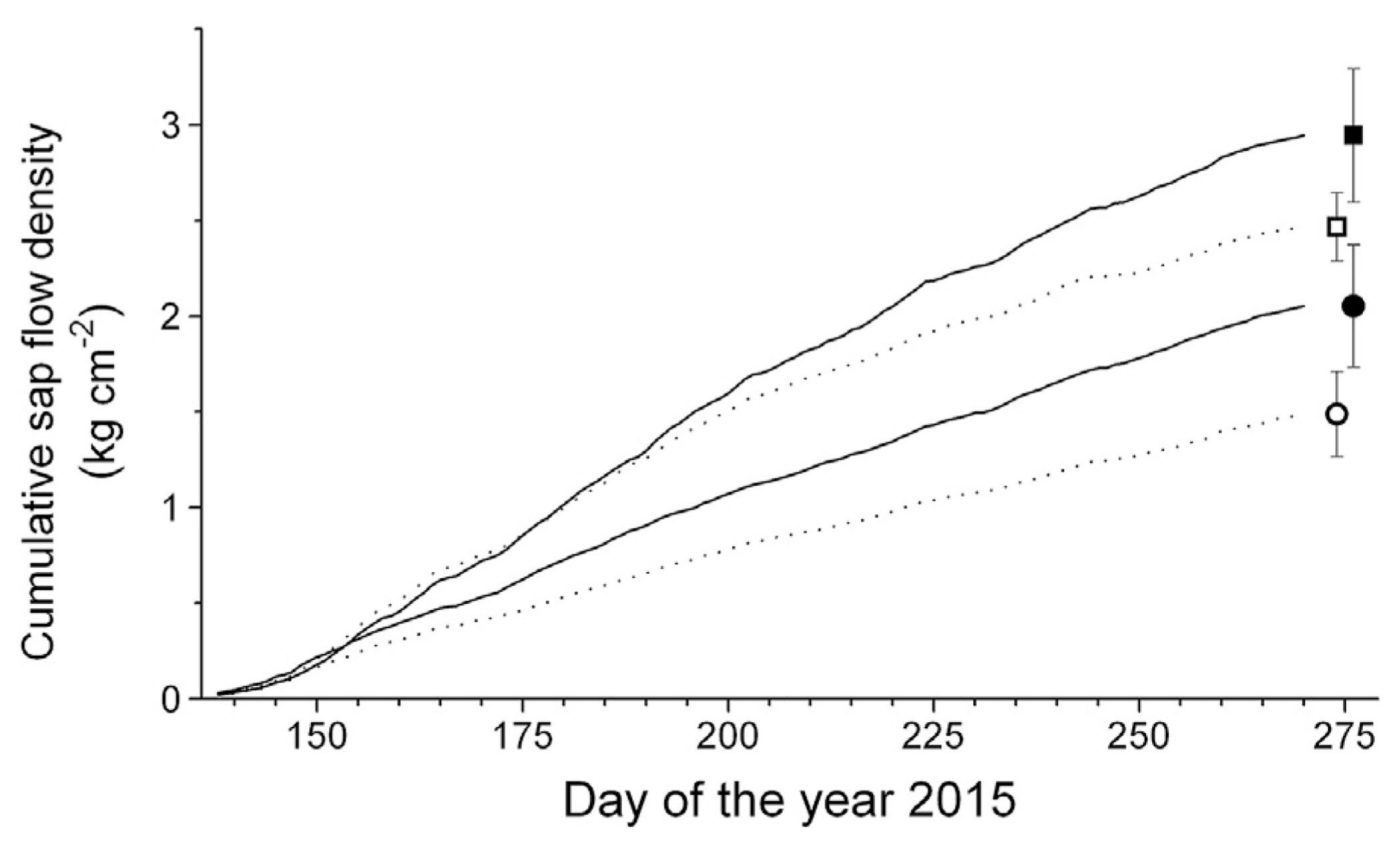
Cumulative xylem sap flow density (Q_s_) after girdling in mid-May (GD doy 138) of the watered and drought-stressed control (continuous lines and closed symbols) and girdled trees (dashed lines and open symbols). Squares and circles denote watered and drought-stressed treatments. Mean standard deviations for the period doy 138–270 are indicated.

Differences in soil water availability between treatments are also reflected in Q_s_ (Fig. 2). Before girdling (doy 77–137) daily mean cumulative Q_s_ values of watered controls (25.1 ± 6.7 g cm^−2^ day^−1^) did not significantly differ from values obtained in drought-stressed controls (23.5 ± 10.2 g cm^−2^ day^−1^), and watered (28.6 ± 10.6 g cm^−2^ day^−1^) and drought-stressed trees (21.1 ± 5.0 g cm^−2^ day^−1^) girdled on doy 138. Hence, girdling was performed before drought stress significantly affected Q_s_. Repeated-measures analysis (post hoc test applying the Bonferroni correction) revealed that after girdling (doy 138–270) daily Q_s_ of drought-stressed saplings was at average 30 ± 1% lower compared to watered trees (*P* < 0.001), consequently cumulative sap flow was significantly reduced (*P* < 0.001; Fig. 2).

### 3.2 Tree phenology and growth

**Fig. 3:**
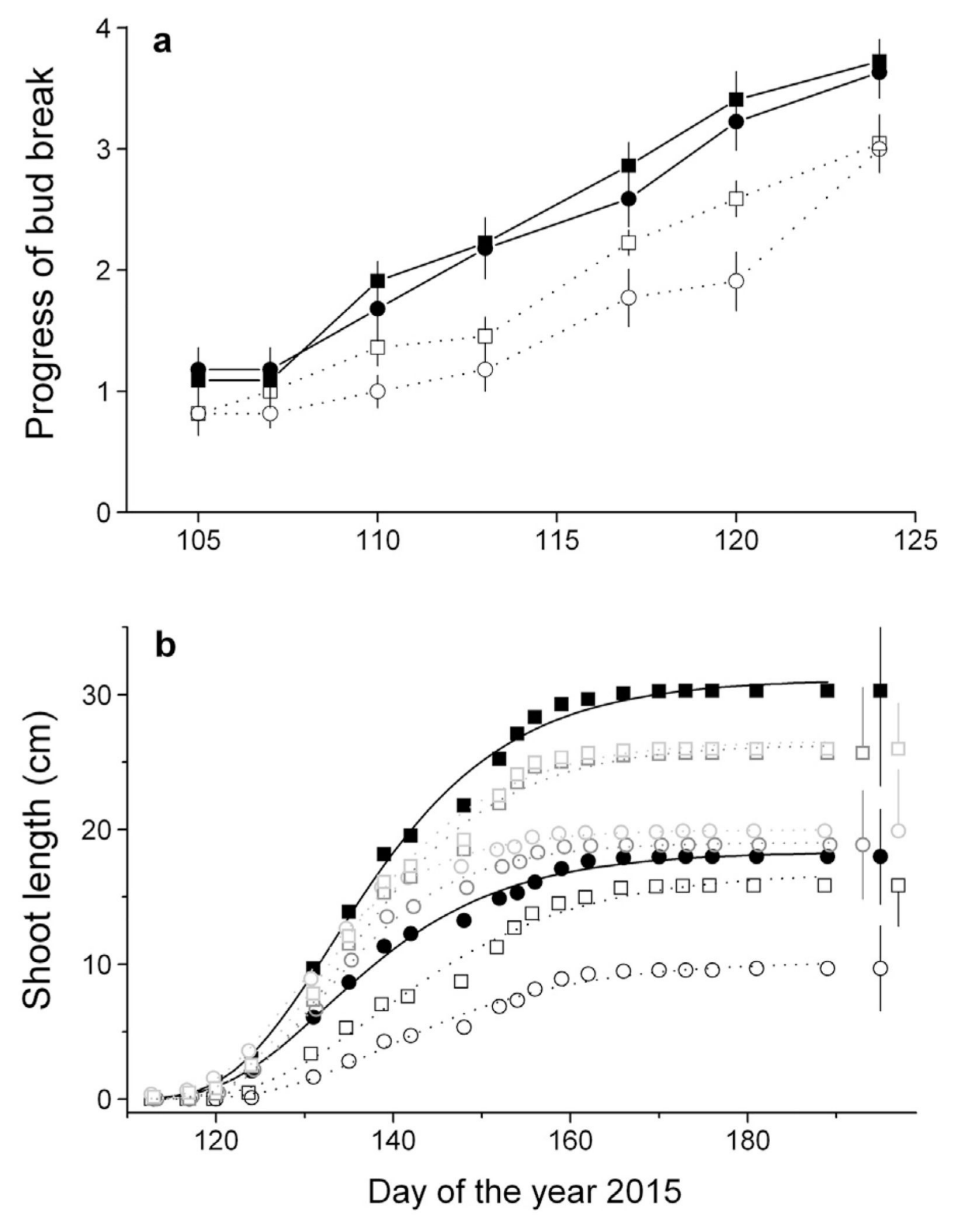
a–b Intra-annual dynamics of bud break (a) and shoot growth (b) of the watered and drought-stressed (closed squares and circles, respectively) control and girdled trees (continuous and dashed lines, respectively). In (b) girdling dates are indicated by black, grey or light grey lines for GDs doy 77, 138 and 190, respectively. Error bars represent standard deviations of samples (a) or the mean standard deviations of all records (b). Short-term variations in intra-annual dynamics of shoot growth were modeled using a Gompertz function (see Materials and Methods for details).

In girdled trees (watered and drought-stressed), bud break was significantly delayed (P ≤ 0.05) compared to the control trees (Fig. 3a). Growth of the terminal leader started in May and already ceased by early June (doy 160; Fig. 3b), i.e., shoot growth duration amounted to *c*. 6 weeks in both treatments. The shoot lengths of the watered and drought-stressed control trees were 30.3 ± 3.4 and 18.0 ± 1.7 cm, respectively. A statistically significant reduction in the leader shoot growth of the trees girdled before growth onset (GD doy 77) was observed, i.e., −47.8% (*P* = 0.001) and −46.1% (*P* = 0.002) in watered and drought-stressed trees, respectively (Table 1). Shoot lengthening was not significantly affected when girdling occurred in mid-May (GD doy 138), and girdling in early July (GD doy 190), when shoot growth has ceased for several weeks, did not break dormancy of already formed buds.

**Fig. 4:**
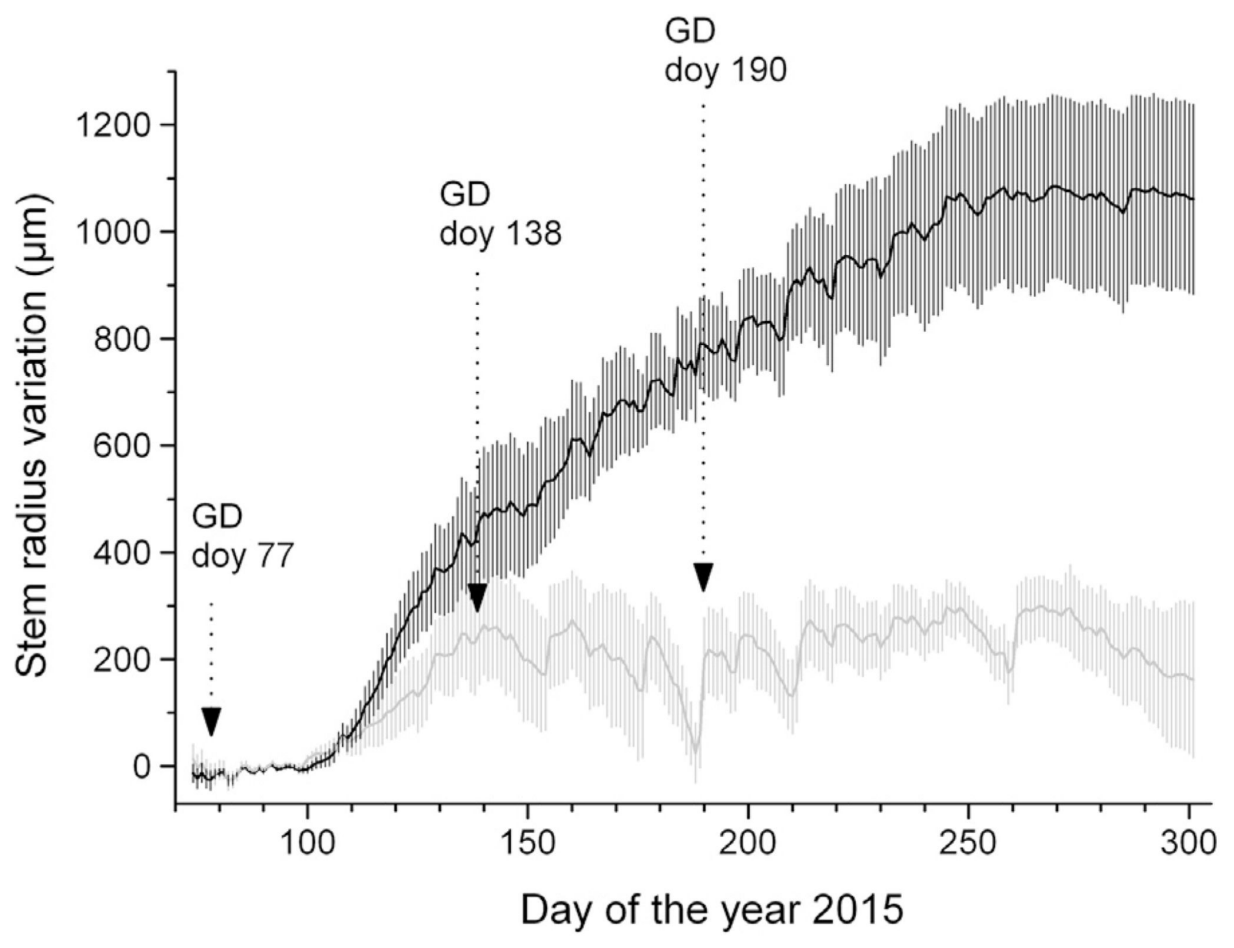
Dendrometer records of non-girdled control trees. Black and grey lines indicate watered and drought-stressed trees, respectively (mean of six trees per treatment; standard deviations are shown). Downward arrows indicate the girdling dates (GD) in other subsets of trees.

The dendrometer records of non-girdled controls trees revealed that the annual increment of drought-stressed trees amounted to *c*. 1/3 of watered trees (Fig. 4). In both treatments RG started in early April (about doy 100) and ceased approximately in early June (doy 160) and mid-September (doy 260) in drought-stressed and watered trees, respectively. Hence, the growing season amounted to *c*. 8 weeks in drought-stressed trees and *c*. 22 weeks in watered trees.

**Fig. 5:**
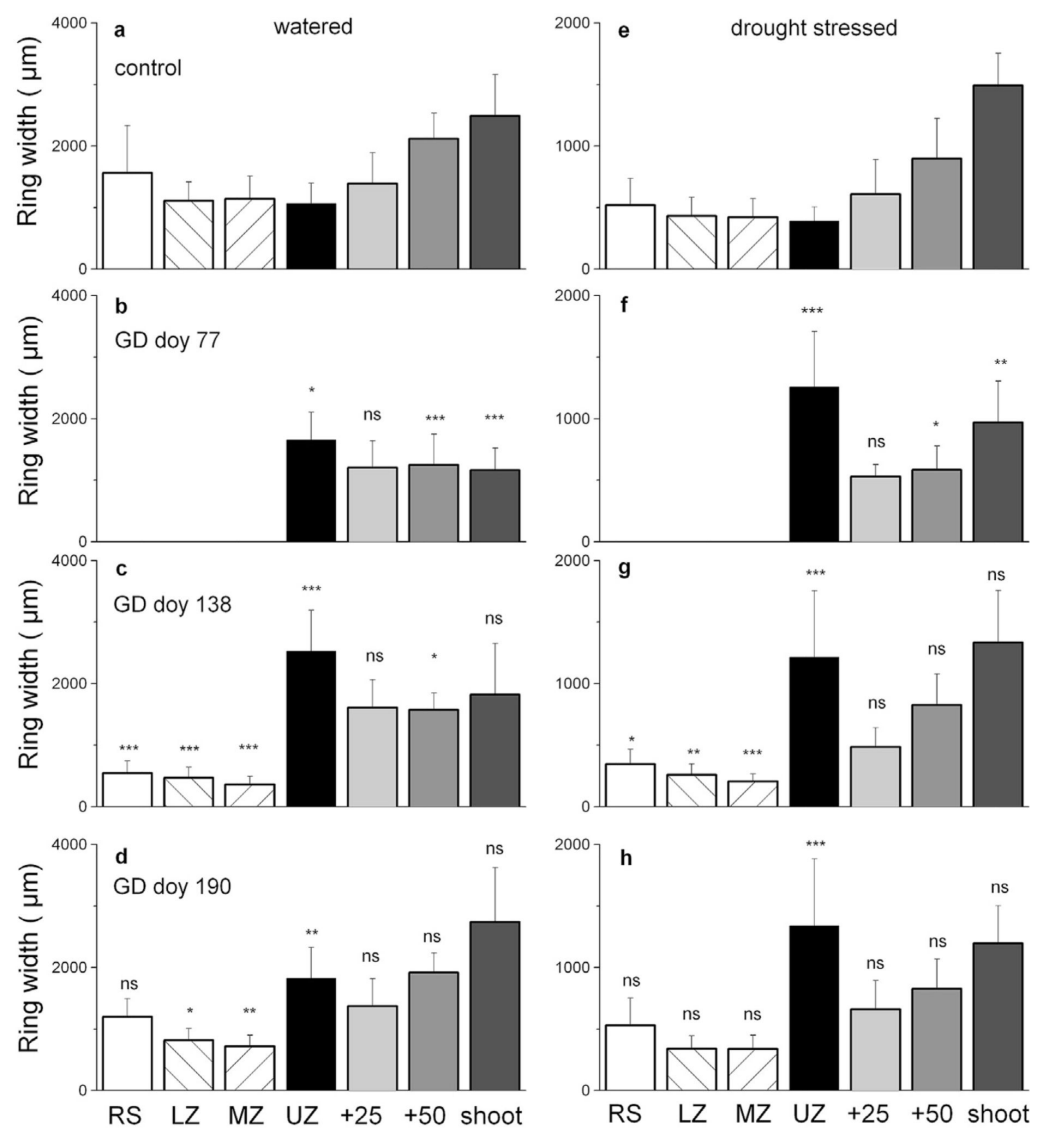
a–h Radial growth along the stem in watered (a–d) and drought-stressed (e–h) control and girdled trees (LZ = lower zone; MZ = middle zone; UZ = upper zone; RS = root/shoot transition; +25 and +50 indicate 25 and 50 cm above UZ, respectively; shoot = current leader). Note that the y-axis differs by a factor of two between the watered and drought-stressed trees. Statistically significant differences among the control and girdled trees are shown. * = *P* < 0.05; ** = *P* < 0.01; *** = *P* < 0.001; ns = not significant. Error bars represent standard deviations.

**Fig. 6:**
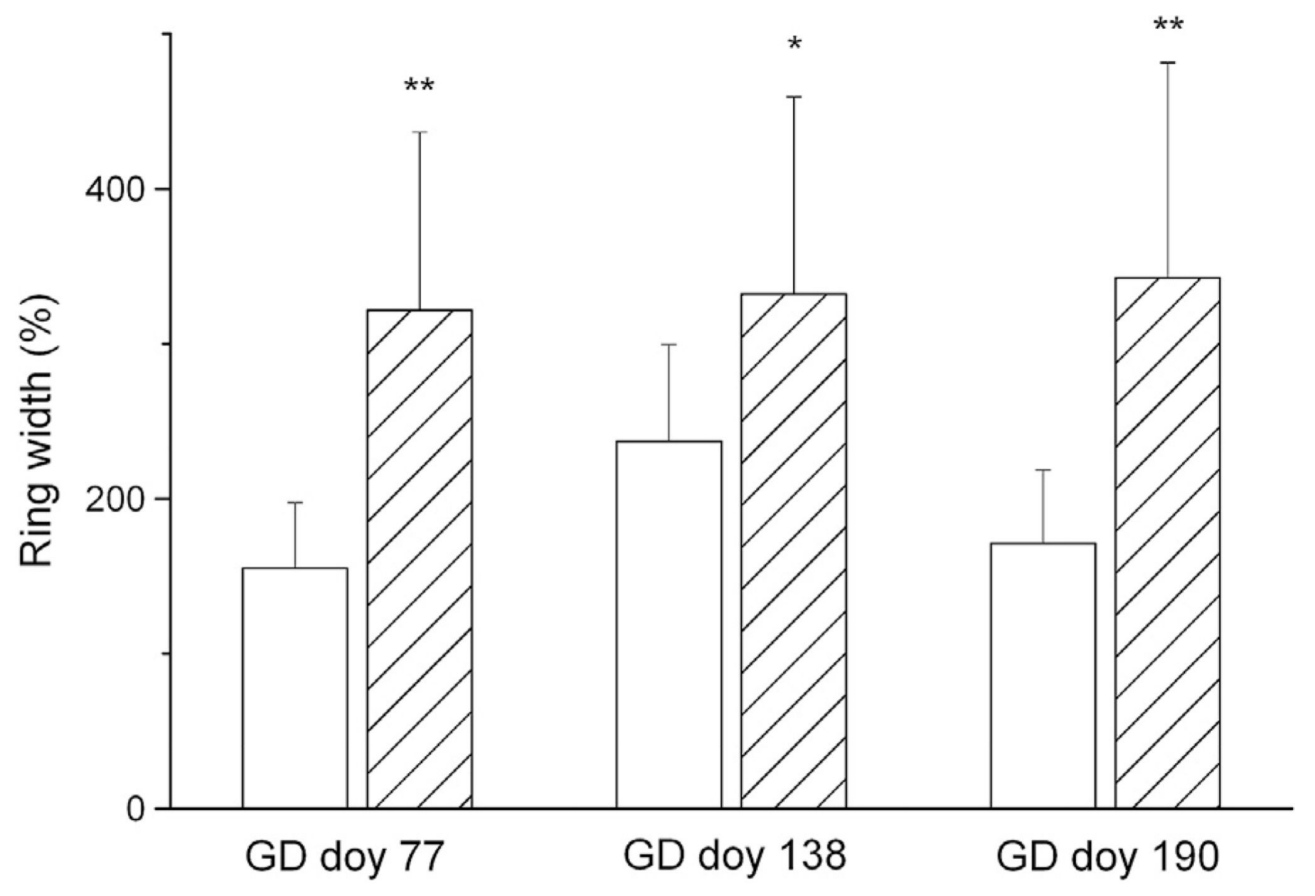
Ring width (%) above girdling (UZ) in watered (open bars) and drought-stressed (hatched bars) trees relative to controls at different girdling dates (GD). Standard deviations and statistical significance of differences among drought-stressed and watered trees are indicated. * = *P* < 0.05; ** = *P* < 0.01.

The effects of girdling on RG are shown in Fig. 5a–h. The main results are: (i) a significant increase in RG of the girdled trees compared to the controls in the UZ, irrespective of water availability and GD. At all GDs the RG increase was significantly more intense in the drought-stressed compared with watered trees and were 3.3 and 1.9-fold higher (mean of all GDs) compared with controls in the drought-stressed and watered trees, respectively (Fig. 6). (ii) A lack of RG between and below the girdle, i.e., in the MZ, LZ and RS, and (iii) a decrease in RG 50 cm above the girdling zone and in the current year leader when girdling occurred before growth onset (GD doy 77). The decrease in RG 50 cm above the girdling zone and in the current leader was consistent with the significant decrease in the current leader growth of the girdled trees (Fig. 3b). (iv) Predominantly no significant change in RG 25 and 50 cm above the girdling zone compared with controls, irrespective of treatment after GDs doy 138 and 190.

**Fig. 7:**
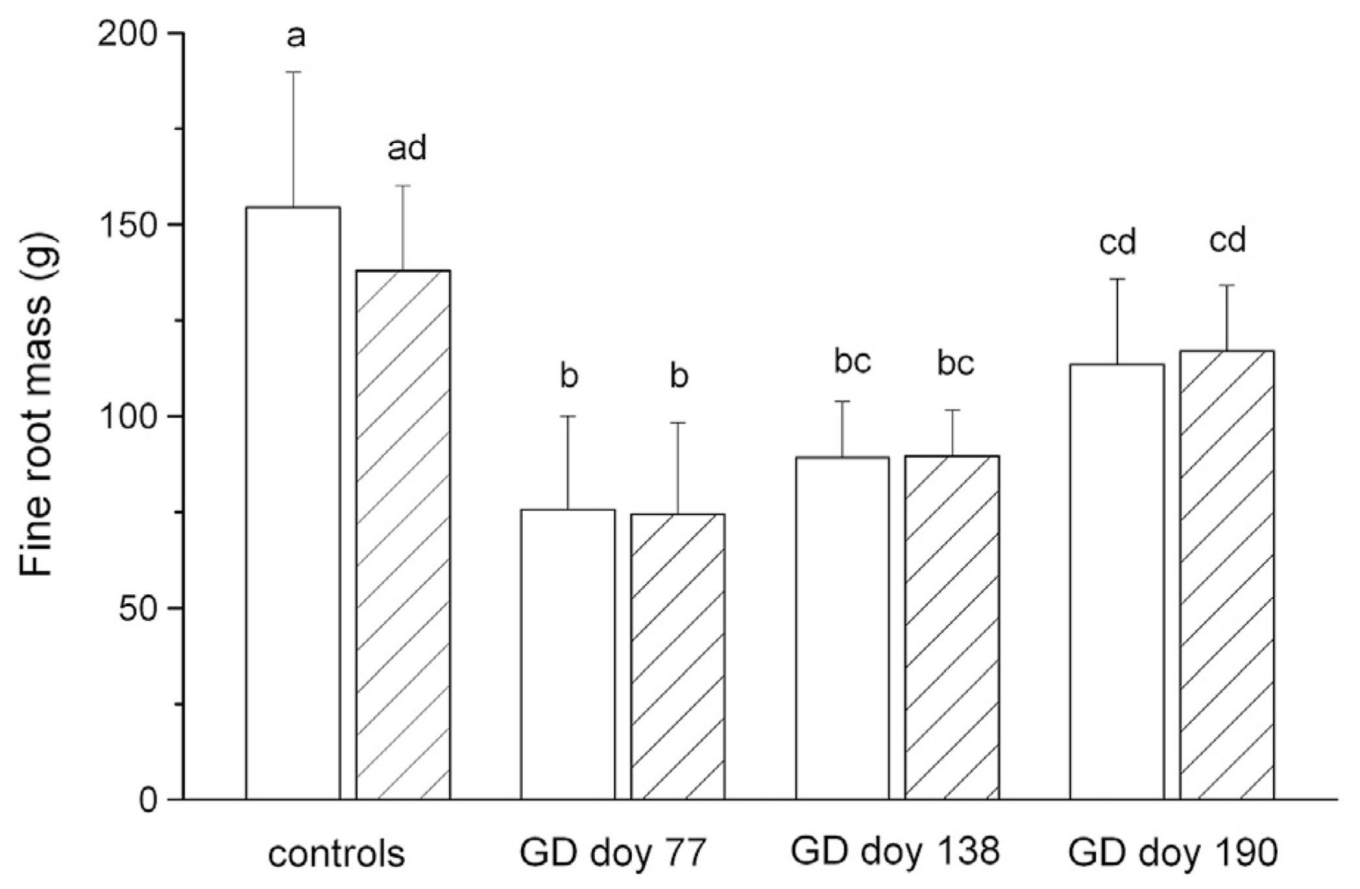
Fine root mass (diameter ≤ 2 mm) of the control and girdled trees at the end of the study period. Watered and drought-stressed trees are indicated by open and hatched bars, respectively. Standard deviations are shown. Statistically significant differences in the mean values of the treatments are indicated by different letters (*P* < 0.01).

The fine root dry mass (root diameter ≤ 2 mm) of the control and girdled trees did not differ significantly among watered and drought-stressed individuals (Fig. 7). Girdling in March (GD doy 77) and mid-May (GD doy 138) significantly (*P* < 0.001) reduced root mass of girdled trees compared to controls (Table 2). Fine root mass determined in trees girdled at doy 190 was significantly higher compared to trees girdled at doy 77, and was significantly reduced in watered trees compared with controls (*P* = 0.004).

### 3.3 Non-structural carbohydrates

**Fig. 8:**
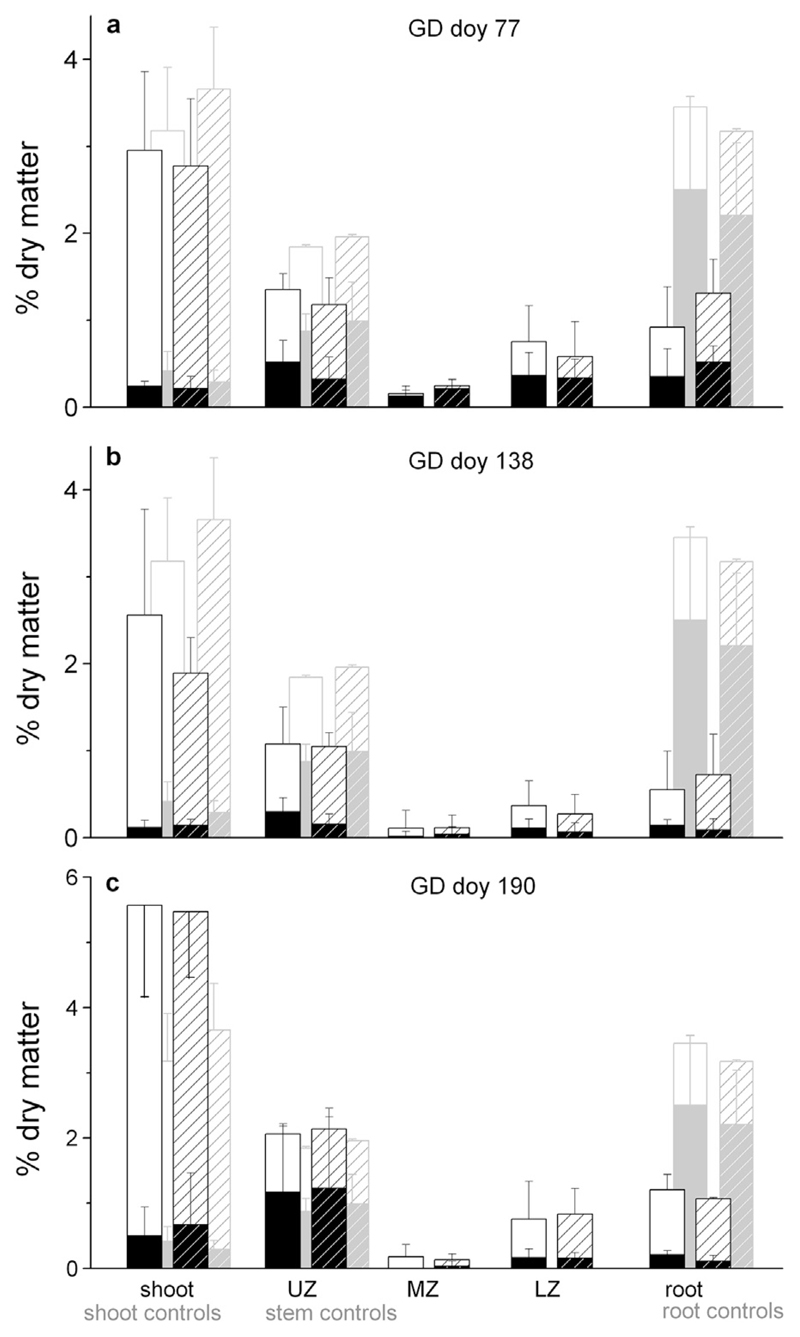
Mean concentration of soluble sugar and starch (open and filled sections of the stacked bars, respectively) at the end of the study period in the current leader (shoot), stem (UZ, MZ and LZ) and coarse roots of watered and drought-stressed control and girdled trees. NSCs in the girdled trees and controls are indicated by black and grey bars, respectively. Open and hatched bars indicate the watered and drought-stressed trees, respectively. For clarity, the position of the grey bars (controls) was slightly shifted laterally. Error bars represent standard deviations.

The main findings pertaining to the effect of double girdling on the NSC content were as follows (Fig. 8, Table 3): (i) no statistically significant differences in the NSC content in the shoot, stem and root among the watered and drought-stressed girdled trees, except total NSC and starch in the root after GD doy 190 (*P* < 0.05), (ii) no accumulation of NSCs directly above the girdling zone (UZ) at all GDs and in both treatments, (iii) the total NSC content in MZ and LZ and in the coarse roots of the watered and drought-stressed girdled trees decreased significantly compared to the control trees at all GDs, whereby in MZ total NSC was lowest of all zones in both treatments and all GDs, (v) the soluble sugar content decreased significantly compared to the controls in the current leader of the drought-stressed girdled trees (GD doy 77 and 138) and significantly increased irrespective of treatment when girdling occurred in July (GD doy 190), and (vi) in the coarse roots, a significant decrease in the starch content (*P* ≤ 0.001) was measured in the watered and drought-stressed trees, irrespective of GD.

In response to girdling tree mortality was observed when girdling occurred prior to growth onset (GD doy 77; all trees died irrespective of water availability between late August and mid-September) and during vigorous radial and shoot growth (GD doy 138; all drought-stressed trees died until the end of September, and four watered trees showed pronounced needle browning at the end of the study period in October). NSC pools measured after tree mortality in response to girdling at doy 138 were not significantly different among treatments (cf. Fig. 8b and data not shown). Tree mortality was not observed in trees girdled in July (GD doy 190) and non-girdled controls.

## 4 Discussion

The main focus of this study was to investigate the effects of interrupted C flow prior to and during the growing season on radial, shoot and root growth and NSC content in watered and drought-stressed *Picea abies* saplings. The direct effect of water availability on tree growth is a well-known phenomenon (e.g., Zahner 1968; Hsiao 1973; Rossi et al., 2009; Rais et al., 2014) and consistent with this, RG and the current leader growth of the drought-stressed, non-girdled trees were significantly reduced compared to the watered trees. However, the fine root mass did not differ significantly among the treatments, which indicates a relatively higher C allocation belowground, i.e., an increase in the root/shoot ratio of drought-stressed trees. Increased C allocation to roots under drought has frequently been reported (for a review, see Brunner et al., 2015; Hagedorn et al., 2016).

Due to blocking of the downward translocation of soluble sugars, accumulation and depletion of carbohydrates above and below the girdling zone, respectively, and depletion of carbohydrate reserves in roots was expected to occur (Högberg et al., 2001; Li et al., 2003; Daudet et al., 2005; Peuke et al., 2005; Maier et al., 2010). Several authors reported that the accumulation of carbohydrates above the girdling zone stimulates RG (Wilson 1968; Noel, 1970; Daudet et al., 2005; De Schepper et al., 2010; De Schepper and Steppe 2011), whereas RG ceases below the girdling zone. Surprisingly, however, irrespective of water availability we determined a statistically significant decrease in total NSCs in UZ of the girdled compared with the control trees, when girdling occurred before growth onset (GD doy 77) or during the intense growth phase (GD doy 138). We explain the lack of accumulation of NSCs in UZ, which was also observed by Daudet et al. (2005) and Cheng et al. (2008), by the significant and striking increase in RG, an increase in maintenance respiration (cf. Domec and Pruyn, 2008) and changes in phenolic and tannin contents as a wound response to girdling. Cambial activity and xylem cell development are considerable energy sinks and depend on a continuous supply of carbohydrates (Hansen and Beck 1994; Oribe et al., 2003; Muller et al., 2011). Hampered NSC supply due to feedback inhibition, i.e., down-regulation of photosynthesis (e.g., Iglesias et al., 2002; Vemmos et al., 2012; Lopez et al., 2015) or the transfer of excess sugars to storage components (Maier et al., 2010; Pantin et al., 2013), can be excluded as we observed no significant decrease in the NSC content in the shoot in response to girdling prior to growth onset in both treatments. Furthermore, it is well established that plants can adjust their C allocation patterns to optimize resource uptake under prevailing environmental constraints (Ericsson et al., 1996; Litton et al., 2007; Nikolova et al., 2011). Hence, it is plausible that the significantly more intense increase in RG in UZ, measured in the drought-stressed compared with watered trees after all GDs, indicates that belowground C allocation limits aboveground growth, causing an early decrease in RG of conifers exposed to drought, which has been reported in previous studies (e.g., Gruber et al., 2010; Oberhuber et al., 2014). Greater C allocation to the root system in drought-stressed trees is supported by the finding that in controls fine root mass did not significantly differ between treatments while aboveground growth (RG and shoot length) was significantly greater in watered compared with drought-stressed trees.

In a modeling study, De Schepper and Steppe (2011) found that changes in turgor pressure due to changes in sugar concentrations were the key driving variable for girdling responses. Similarly, Pantin et al. (2013) suggest that plants use soluble carbohydrates to buffer expansive growth against fluctuations in water availability. The requirement of adequate cell turgor for cell division and cell enlargement has been reported, e.g., by Zweifel et al. (2006), Rossi et al. (2013) and Deslauriers et al. (2016). Hence, the more than 3-fold and about 2-fold increase in RG in UZ of the drought-stressed and watered trees, respectively, might be due to short-term accumulation of osmotically active sugars, which increase osmotic pressure according to the van't Hoff equation (Jones, 1992) and cause more water to be drawn into the cambial zone, favoring cell division and expansion.

Irrespective of GD, inhibition of RG occurred between (MZ) and below the girdling zone (LZ, RS), which is in accordance with previous findings (Maier et al., 2010; Maunoury-Danger et al., 2010; De Schepper and Steppe, 2013) and also indicates that starch reserves are primarily used for maintenance processes. In addition, an uninterrupted phloem connection between the leaf and shoot at the start of the growing season seems to be a prerequisite for RG to occur. Millard et al. (2007) and Sala et al. (2012) suggested that a considerable fraction of starch becomes sequestered rather than stored, which could explain why starch pools in MZ were not fully depleted during the study period (March through October), when girdling occurred prior to growth onset. The finding that almost full depletion of starch content in MZ occurred when girdling was applied during the growing season can be explained by ongoing RG prior to girdling. Decreasing levels of starch during the growing season were reported in tree stems by e.g., Hoch et al. (2003), Deslauriers et al. (2009) and Gruber et al. (2012) and were also found in this study (data not shown).

In *Picea abies* and other conifers, high levels of indole-3-acetic acid (IAA) are found in cambial tissues during dormancy (e.g., Sundberg et al., 1990; Savidge, 1991; Eklund et al., 1998), and cambial cell division can be initiated during the quiescent stage by localized heating of the stem, independent of the growth of new shoots and the development of buds (e.g., Oribe and Kubo, 1997; Oribe et al., 2001; Gričar et al., 2006). Consequently, a lack of cambial activity below the girdling zone might indicate that sugar signaling, which has been known in promoting cell division for many years (Uggla et al., 2001; Smith and Stitt, 2007; Eveland and Jackson, 2012; Lastdrager et al., 2014) or current photosynthates (Hansen and Beck, 1994; Oribe et al., 2003) are necessary for RG to occur. Because phloem blockage stopped cambial activity below the girdling zone, non-significant differences in RG below the girdling zone of drought-stressed (GD doy 190) and control trees indicate completed RG at this time, which is consistent with dendrometer records. Hence, in drought-stressed trees cambial reactivation occurred in UZ after girdling at doy 190. In *Picea abies* a wound induced reactivation of the cambium after the cessation of its regular activity was also reported by Gričar et al. (2007) and related to hormonal unbalance. We suggest that osmotically active C compounds, which are necessary to generate water turgor pressure during cell expansion (Deslauriers et al., 2009; Simard et al., 2013; Steppe et al., 2015) and release the hydromechanical limitation of growth (Pantin et al., 2013), most likely temporarily accumulated above the girdling zone and together with a ‘signaling compound’ (sugar- and/or auxin based) induced tremendous RG, even after regular cambial activity and shoot growth have ceased in drought-stressed trees.

Although no RG occurred in MZ, the NSC reserves were almost completely used, which is explained by respiration of the living stem cells and the intensive production of resin in MZ in response to wounding above and below this zone. Maunoury-Danger et al. (2010) and De Schepper and Steppe (2011) also suggested that after girdling, more C is directed to secondary metabolism as a wounding response. The undisrupted phloem connection between LZ and the root stores did not substantially increase the NSC content in LZ, which most likely indicates a lack of acropetal C transport from the root reserves to the shoot. The consistently measured NSC content in dead girdled trees in the stem and coarse roots supports the view that some NSCs may represent C sequestration rather than storage (Millard et al., 2007). The same explanation was put forward by Li et al. (2015), who found abundant NSC stores remaining in roots of dead girdled trees.

The upper bole is closer to the active growth region of the crown, i.e., this section is the first to receive new sugars and hormones before they are transported to other tree parts (for a review, see Bhalerao and Fischer, 2017), and the shoot apex has a higher ranking priority than the stem cambium as a sink for C (Minchin and Lacointe, 2005). However, RG 50 cm above UZ and shoot lengthening significantly decreased in the girdled (GD doy 77) compared to the control trees in both treatments. Similarly, a decrease in shoot growth after girdling was reported by Cutting and Lyne (1993), McFadyena et al. (2013) and Tombesi et al. (2014). In the present study, (i) soluble sugars were the main carbohydrate found in the current leader of the girdled trees, indicating there was no C shortage, and (ii) the shoot length was significantly longer in the watered compared to the drought-stressed control trees. These results suggest that the retarded progression of bud break and the reduction of shoot lengthening and RG in the current leader of the trees girdled prior to growth onset were caused by a decrease in water availability at the top of the tree, which is corroborated by reduced xylem sap flow in girdled trees of both treatments and more severe tree mortality in drought-stressed girdled trees. Decreases in sap flow and stem water potential due to girdling are frequently reported and are explained by decreases in hydraulic conductance due to the diffusion of air into the xylem under high evaporative demand and/or reduced root activity or by decreases in conductance caused by the blockage of C transport from the canopy (Zwieniecki et al., 2004; Sellin et al., 2013; Tombesi et al., 2014; Lopez et al., 2015). The lack of significant differences in the progression of bud break between the watered and drought-stressed control trees was related to the use of stem water reserves at the start of the growing season. This explanation is corroborated by findings presented by Oberhuber et al. (2015), who reported that the use of internal stem water reserves makes *Picea abies* less dependent on current water availability in the soil. The lack of significant reduction of shoot lengthening when girdling occurred during the period of shoot growth (GD doy 138) is consistent with findings of Tombesi et al. (2014), who reported that the earlier girdling occurred, the larger the reduction in midday stem water potential and the stronger the effect on shoot growth.

## 5 Conclusion

In accordance with our hypothesis, this study demonstrates that under the conditions of our experimental manipulation, i.e., double girdling and drought stress, water and C availability control aboveground and belowground growth of *Picea abies* saplings. The findings (i) that irrespective of GD the altered C availability consistently stimulated RG above girdling (which contradicts our hypothesis of different RG response to girdling during the growing season) and (ii) that the RG increase in girdled trees was significantly more intense in the drought-stressed compared to watered treatment, indicate a pronounced C requirement of the root system particularly under drought. Hence, our results support the idea that early achievement of the maximum RG rate reported for several coniferous species exposed to drought (Oberhuber et al., 2014) is due to an early switch of C allocation to belowground organs to sustain adequate tree water status. Because physical blockage of the phloem also affects the transport of growth substances, possibly leading to hormonal imbalances, whether girdling induces physiological changes unrelated to C status needs to be evaluated.

## Figures and Tables

**Table 1:** Statistical significance (*P*-values) of differences in shoot growth between control and girdled trees growing under different soil water availability (doy = day of the year, GD = girdling date). Repeated measure analysis with post-hoc multiple comparisons (Bonferroni correction) was applied. Significant *P*-values (*P* ≤ 0.05) are in bolt.

	Watered				Drought-stressed		
	GD doy 77	GD doy 138	GD doy 190		GD doy 77	GD doy 138	GD doy 190
Watered control	0.001	0.315	0.299	Drought control	0.002	0.532	0.248
Watered GD doy 138	**0.002**			Drought GD doy 138	**0.001**		
Watered GD doy 190	**0.0005**	0.845		Drought GD doy 190	**0.0005**	0.561	

**Table 2:** Statistical significance (*P*-values) of differences in fine root dry mass between control and girdled trees growing under different soil water availability. Repeated measure analysis with post-hoc multiple comparisons (Bonferroni correction) was applied. Significant *P*-values (*P* < 0.01) are in bolt (doy = day of the year, GD = girdling date).

	Watered control	Drought control	Watered GD doy 77	Drought GD doy 77	Watered GD doy 138	Drought GD doy 138	Watered GD doy 190
Drought control	1.000						
Watered GD doy 77	0.000	0.000					
Drought GD doy 77	**0.000**	**0.000**	1.000				
Watered GD doy 138	**0.000**	**0.000**	1.000	1.000			
Drought GD doy 138	**0.000**	**0.000**	1.000	1.000	1.000		
Watered GD doy 190	**0.004**	0.625	**0.002**	**0.002**	0.127	0.172	
Drought GD doy 190	**0.007**	0.956	**0.001**	**0.001**	0.212	0.279	1.000

**Table 3:** Statistical significance (*P*-values; Mann-Whitney *U* test) of differences in total non-structural carbohydrates (NSC), soluble sugars and starch in shoot, stem and root in control vs. watered and drought-stressed girdled trees. Stem sections were sampled above (UZ), between (MZ) and below (LZ) double girdling. *P*-values <0.05 are printed in bolt (doy = day of the year, GD = girdling date).

		GD doy 77	GD doy 138	GD doy 190
*Watered*				
Total NSC	shoot	0.654	0.114	0.000
	UZ	0.004	**0.003**	0.165
	MZ	**0.000**	**0.000**	**0.000**
	LZ	**0.000**	**0.000**	**0.002**
	root	**0.000**	**0.000**	**0.000**
Soluble sugars	shoot	0.971	0.251	**0.000**
	UZ	0.133	1.000	0.468
	MZ	**0.000**	**0.000**	**0.000**
	LZ	**0.005**	**0.000**	**0.043**
	root	**0.010**	**0.029**	0.426
				
Starch	shoot	0.132	**0.000**	0.941
	UZ	**0.006**	**0.000**	0.863
	MZ	**0.000**	**0.000**	**0.000**
	LZ	**0.000**	**0.000**	**0.000**
	root	**0.000**	**0.000**	**0.000**
*Drought-stressed*				
Total NSC	shoot	0.035	0.000	**0.010**
	UZ	**0.001**	**0.000**	0.468
	MZ	**0.000**	**0.000**	**0.000**
	LZ	**0.000**	**0.000**	**0.000**
	root	**0.000**	**0.000**	**0.000**
				
Soluble sugars	shoot	**0.043**	**0.000**	**0.002**
	UZ	0.238	0.197	0.468
	MZ	**0.000**	**0.000**	**0.000**
	LZ	**0.002**	**0.000**	**0.002**
	root	0.863	0.365	0.478
				
Starch	shoot	0.349	**0.007**	0.918
	UZ	**0.001**	**0.000**	0.666
	MZ	**0.000**	**0.000**	**0.000**
	LZ	**0.000**	**0.000**	**0.000**
	root	**0.000**	**0.000**	**0.000**
